# Molecular detection and typing of methicillin-resistant *Staphylococcus aureus* and methicillin-resistant coagulase-negative staphylococci isolated from cattle, animal handlers, and their environment from Karnataka, Southern Province of India

**DOI:** 10.14202/vetworld.2019.1760-1768

**Published:** 2019-11-11

**Authors:** Nimita Venugopal, Susweta Mitra, Rituparna Tewari, Feroze Ganaie, Rajeswari Shome, Habibur Rahman, Bibek R. Shome

**Affiliations:** 1 ICAR-National Institute of Veterinary Epidemiology and Disease Informatics, Bengaluru, Karnataka, India; 2 Department of Microbiology, Jain University, Bengaluru, Karnataka, India; 3 School of Basic and Applied Sciences, Dayananda Sagar University, Bengaluru, Karnataka, India; 4 Department of Medicine, Division of Pulmonary/Allergy/Critical Care, University of Alabama at Birmingham, Alabama, USA; 5 International Livestock Research Institute, New Delhi, India

**Keywords:** *mec*A/*mec*C gene, methicillin resistance, methicillin-resistant coagulase-negative staphylococci, methicillin-resistant *Staphylococcus aureus*

## Abstract

**Background and Aim:**

Methicillin-resistant staphylococci are among the emerging pathogens which have become a threat to both human and animal health. The present investigation intended to examine the occurrence and the molecular characteristics of methicillin-resistant *Staphylococcus aureus* (MRSA) and methicillin-resistant coagulase-negative staphylococci (MRCoNS) recovered from cattle, its handlers, and their environment.

**Materials and Methods:**

A total of 666 specimens were subjected to culture method and genus-specific polymerase chain reaction (PCR) for the identification of *Staphylococcus*. Methicillin resistance was substantiated by PCR identification of *mec*A and *mec*C resistance determinants. Species-specific identification of *mec*A positive isolates was conducted by multiplex PCR. The unidentified species were deciphered by 16S rRNA gene sequencing approach. The *mec*A positive isolates were further characterized by staphylococcal cassette chromosome *mec* (SCC*mec*) typing and multilocus sequence typing (MLST).

**Results:**

Duplex PCR identified 728 *Staphylococcus* isolates, of which 66 (9%) were positive for *mec*A gene. MRSA constituted 24% of the total *mec*A positive isolates. Among MRCoNS, *Staphylococcus epidermidis* (42%), and *Staphylococcus haemolyticus* (11%) were the most common species identified. Overall, 47% of the *mec*A positive isolates belonged to SCC*mec* type V. MLST analysis showed eight different sequence types (STs) among MRSA isolates of which five were novel STs. Among methicillin-resistant *S. epidermidis*, 19 different STs were found, of which nine novel STs were detected.

**Conclusion:**

The increase in the prevalence of *mec*A positive staphylococci, especially MRCoNS in cattle is a great concern in view of their transmission potential. Hence, continuous monitoring and molecular characterization of methicillin-resistant staphylococci should be elucidated in human and animal sectors so as to prevent the spread of these resistant pathogens.

## Introduction

Over 50 species of the *Staphylococcus* genus have been described until now [1]. *Staphylococcus aureus*, a coagulase-positive species is a well-recognized nosocomial pathogen in both human and animal medicine. It is the most important pathogen recognized for intermittent infections and outbreaks [2], whereas coagulase-negative staphylococci (CoNS) signifying preponderance of species have been thought to be saprophytic or rarely pathogenic [3]. Over the past few decades, studies have revealed CoNS as the prime etiological agent of a series of infectious processes, ranging from hospital-acquired infection to livestock bacterial sepsis and mastitis [4].

*S. aureus* has a distinctive capacity to swiftly develop resistance to almost any antibiotics arriving into clinical practice. Methicillin resistance that demonstrates resistance to all available β-lactam antibiotics was initially described in 1961, which denoted the emergence of methicillin-resistant *S. aureus* (MRSA) [5]. It is presumed that determinants of methicillin resistance had emerged in CoNS and then disseminated horizontally among staphylococci [6]. Methicillin resistance is conferred by an altered penicillin-binding protein 2a encoded by *mec*A gene sited on a mobile genetic element called staphylococcal cassette chromosome (SCC) [7]. In 2011, a different *mec*A homolog, designated as *mec*C (previously *mec*A_LGA251_) was identified in bovine milk and human clinical specimens in different parts of the world [8-10]. The *mec*C has about 70% comparability with *mec*A at the nucleotide level and 63% identity at the amino acid level. The *mec*A_LGA251_ was discovered to carry a novel SCC*mec* element Type XI [11]. Since SCC*mec* element is the known vector to transfer the *mec*A and *mec*C gene among *Staphylococcus* species or between animals and humans and their environment; hence, it is imperative to detect the SCC*mec* type.

Over the years, the occurrence of MRSA has been progressively increasing across the globe [12]. In India, MRSA is recognized endemic with the prevalence rate of MRSA and methicillin-resistant CoNS (MRCoNS) varying from 17 to 70% and 22 to 73%, respectively [13,14]. Recent studies report the recovery of MRCoNS from diverse sources including medical devices, healthy humans, ambulatory patients, and bloodstream infections. The appearance of MRCoNS in animals was first explained in chicken [15]. However, MRSA and MRCoNS were also found in different animal species with clinical infections. Studies have detailed *Staphylococcus epidermidis* and *Staphylococcus haemolyticus* as the foremost pathogens engaged with a wide range of infections of humans and animals. Limited data are accessible on the prevalence and genomic characteristics of MRSA and MRCoNS from animal sector; thereby, it is likely to miss other spillover events of these pathogens between livestock and humans or vice versa [16].

Surveillance of MRSA and MRCoNS infections in both human and animal health-care settings is critical due to the changing epidemiological profile of organisms. Precise and quick detection of these pathogens allows application of efficient antimicrobial therapy and preventive infection control strategies [17]. The increasing significance of MRSA and MRCoNS serves the justification for more accurate species identification to allow the exact determination of host-pathogenic possibility of each of the different species [18]. The current study aimed to investigate the occurrence and characterize MRSA and MRCoNS recovered from cattle, animal handlers, and their environment.

## Materials and Methods

### Ethical approval

The study was approved by the Institutional Ethics Committee of ICAR-NIVEDI. All applicable international, national, and institutional guidelines for the animal’s care were followed during the sample collection.

### Specimen collection

A total of 666 specimens were collected between 2015 and 2017 from five organized cattle farms (Kanakapura [12.9428°N, 77.5779°E], Kagalipura [12.7995°N,77.5101°E], Agara [12.9231°N, 77.6465°E], Bidadi [12.7968°N, 77.3839°E], and Arehalli [12.9117°N, 77.5412°E]), and eight unorganized cattle herds (Kodihalli [12.9654°N, 77.6493°E], Kadabagere [12.9969° N, 77.4331° E], Ramagondanahalli [12.9558°N, 77.7409°E], Hasanghatta [13.1500°N, 77.4900°E], Tapasihalli [13.3907°N, 77.6859°E], Doddaballapur [13.2895°N, 77.5342°E], Jakkanahalli [13.1783869 °N, 77.3467876°E], and Yediyur [12.9312 °N, 77.5723°E]) located in and around Bengaluru, India. The samples comprised cattle milk (n=371), cattle nasal swabs (n=109), extramammary site (n=90), wound samples from cattle (n=30), animal handlers hand swabs (n=32), and environmental swabs (n=34). The environmental samples included feed trough (n=13), floor of cattle shed (n=15), milking machine (n=4), and supplied water (n=2).

### Isolation and tentative identification of *Staphylococcus* spp.

The specimens were inoculated into Brain Heart Infusion (BHI) Broth and transported to the Microbial Pathogenesis and Pathogen Diversity Laboratory, ICAR-NIVEDI, Bengaluru, India, within 2 h. Each sample was initially cultured on staphylococcus agar 110 (S110) (HiMedia, Mumbai) and incubated at 37°C for 24 h. Pure culture of isolates was obtained by subculture on BHI agar (HiMedia, Mumbai). *Staphylococcus* was identified based on colony characteristics, pigment production, Gram staining, catalase, and oxidase tests as per the standard protocol [19].

### DNA extraction

The genomic DNA from the staphylococci was extracted using the QIAamp DNA Mini Kit (Qiagen, Duesseldorf, Germany) as per manufacturer’s recommendations. NanoDrop 2000c (Thermo Fisher Scientific Inc., Waltham, MA, USA) was used to determine the purity and concentration of the extracted DNA.

### Duplex polymerase chain reaction (PCR) for detection of genus *Staphylococcus* and *mecA* gene

The extracted DNA from all the Gram-positive isolates was subjected to our in-house duplex PCR assay for the simultaneous detection of genus *Staphylococcus* and methicillin resistance determinant (*mec*A gene) (Table-1). The primers were synthesized based on the previously published sequences [20,21]. Duplex PCR assay was performed in a 25 µL reaction volume containing 1X PCR buffer, 1.5 U DNA *Taq* polymerase, 2mM MgCl_2_, and 200 µM deoxynucleotide triphosphates; (Fermentas, Glen Burnie, MD, USA), 0.6 μM and 0.5 μM of *Staphylococcus* genus and *mec*A specific primers, respectively, and 50 ng template DNA. The PCR cycling conditions comprised an initial denaturation step at 94°C for 5 min, followed by 30 cycles of denaturation at 94°C for 30 s, annealing at 56°C for 30 s, extension at 72°C for 45 s, and final extension step at 72°C for 5 min. The ATCC 33591 MRSA reference strain was used for the optimization of the assay.

**Table-1:** Details of primers used for the identification and characterization of MRSA and MRCoNS.

S. No.	PCR	Primers	Sequence	Amplicon size	Annealing temp	References
1.	Duplex PCR	16S rRNA F*	GTGATCGGCCACACTGGA	842 bp	56°C	[20] [21]
		16S rRNA R^†^	CAACTTAATGATGGCAACTAAGC			
		*mec*A F*	ACGAGTAGATGCTCAATATAA	292 bp		
		*mec*A R^†^	CTTAGTTCTTTAGCGATTGC			
2.	mecC PCR	*mec*LGA251 F*	GCTCCTAATGCTAATGCA	304 bp	50°C	[22]
		*mec*LGA251 R^†^	TAAGCAATAATGACTACC			
3.	Multiplex PCR	*S. chromogenes* F*	GCGTACCAGAAGATAAACAAACTC	222 bp	60°C	[23]
		*S. chromogenes* R^†^	CATTATTTACAACGAGCCATGC			
		*S. haemolyticus* F*	CAAATTAAATTCTGCAGTTGAGG	531 bp		
		*S. haemolyticus* R^†^	GGCCTCTTATAGAGAGACCACATGTTA			
		*S. epidermidis* F*	AAGAGCGTGGAGAAAAGTATCAAG	130 bp		
		*S. epidermidis* R^†^	TCGATACCATCAAAAAGTTGG			
		*S. sciuri* F*	GATTCCGCGTAAACGGTAGAG	306 bp		
		*S. sciuri* R†^†^	CATCATTTAATACTTTAGCCATTG			
		*S. aureus* F*	AGCGAGTCTGAATAGGGCGTTT			
		*S. aureus* R^†^	CCCATCACAGCTCAGCCTTAAC			
4.	Partial 16S rRNA gene sequencing	S-seq F*	GCGGACGGGTGAGTAACAC	974 bp	60°C	[24]
		S-seq R^†^	GACGACAACCATGCACCAC			
5**.**	mPCR1-ccr typing	*mec*A2 F*	TGCTATCCACCCTCAAACAGG	286 bp	56°C	[25]
		*mec*A2 R^†^	AACGTTGTAACCACCCCAAGA			
		ccrB F	ATTGCCTTGATAATAGCCTTCT	695 bp		
		α1 R^†^	AACCTATATCATCAATCAGTACGT			
		α2 R^†^	TAAAGGCATCAATGCACAAACACT	937 bp		
		α3 R^†^	AGCTCAAAAGCAAGCAATAGAAT	1791 bp		
		ccr A4 F*	GTATCAATGCACCAGAACTT	1287 bp		
		ccr B4 R^†^	TTGCGACTCTCTTGGCGTTT			
		ccr C F*	CGTCTATTACAAGATGTTAAGGATAAT	518 bp		
		ccr C R^†^	CCTTTATAGACTGGATTATTCAAAATAT			
	mPCR2-mec typing	Class A*mec* F*	CATAACTTCCCATTCTGCAGATG	1963 bp	60°C	
		Class B*mec* F*	ATGCTTAATGATAGCATCCGAATG	2827 bp		
		Class C*mec* F*	TGAGGTTATTCAGATATTTCGATGT	804 bp		
		Class A/B/C *mec* R^†^	ATATACCAAACCCGACAACTACA			

* F=Forward primer sequence;

† R=Reverse primer sequence; In mPCR1-ccr typing, ccrB was used as a common forward primer with α1, α2, and α3 reverse primers; In mPCR2-mec typing, a common reverse primer (Class A/B/C *mec*R) was used with three different forward primers (Class A *mec*, Class B *mec*, and Class C *mec*)

### Uniplex PCR for identification of *mecC* gene

Uniplex PCR based testing of *mec*C gene (a homolog of *mec*A gene) was carried out for all the staphylococcal isolates (Table-1) [22]. PCR assay was done in a 15 μL reaction volume containing 1X PCR ready master mix (0.025U DNA *Taq* polymerase in reaction buffer, 2 mM MgCl_2_, and 200 mM deoxynucleotide triphosphates [Fermentas, Glen Burnie, MD, USA]), 0.5 μM of *mec*C specific primers and 50 ng of extracted DNA. The PCR cycling conditions comprised an initial denaturation step at 95°C for 2 min, followed by 30 cycles of denaturation at 94°C for 30 s, annealing at 50°Cfor 30 s, extension at 72°C for 30 s, and final extension step at 72°C for 4 min.

### Species-specific identification

All *mec*A/*mec*C positive *Staphylococcus* isolates were deciphered to species level by species-specific multiplex PCR targeting five major *Staphylococcus* spp., namely, *S. aureus*, *S. epidermidis*, *S. haemolyticus*, *Staphylococcus chromogenes*, and *Staphylococcus sciuri* (Table-1) [23].

### Partial 16SrRNA gene sequencing of *Staphylococcus* spp.

The unidentified *mec*A/*mec*C positive strains were subjected to partial 16S rRNA gene sequencing for species-specific identification. The primer pair used was flanking five hypervariable regions (V2, V3, V4, V5, and V6) of 16S rRNA to amplify a 974 bp fragment (Table-1). A simplex PCR assay for amplification of DNA was performed in 50 µL reaction volume containing 0.5 μM of 16S rRNA specific primers. The reaction mixture and the cycling conditions were similar as previously published by Shome *et al*. [24]. The PCR amplicons were sequenced in bi-direction by Sanger sequencing approach at Eurofins, Bengaluru, India.

### Molecular characterization by *SCCmec* typing

The *mec*A/*mec*C positive strains were subjected to PCR-directed SCC*mec* typing as earlier detailed by Kondo *et al*. [25]. The strategy comprised two multiplex PCR assays mPCR-1 for *ccr* typing and mPCR-2 for *mec* class typing (Table-1).

### Multilocus sequence typing (MLST)

MRSA and methicillin-resistant *S. epidermidis* (MRSE) were characterized by MLST analysis. Specifically, the MLST analysis was conducted by sequencing fragments of seven housekeeping genes: *arc*C, *aro*E, *glp*F, *gmk*, *pta*, *tpi*, and *yqi*L for *S. aureus* and *arc*C, *aro*E, *gtr*, *mut*S, *pyr*, *tpi*, and *yqi*L for *S. epidermidis*. Allele number and sequence types (STs) were assigned using the *S. aureus* and *S. epidermidis* MLST websites (https://pubmlst.org/saureus/and https://pubmlst.org/sepidermidis/).

## Results

### Molecular detection of *Staphylococcus* and methicillin resistance

**Figure-1:**
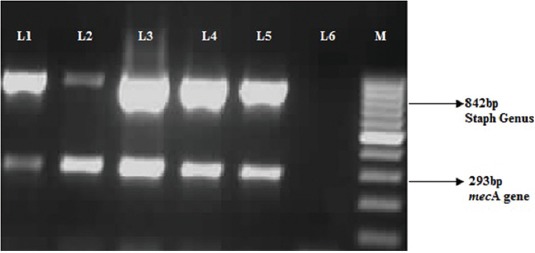
Duplex polymerase chain reaction for simultaneous detection of genus *Staphylococcus* and *mec*A gene. L1=Positive control (ATCC 33591); L2-L5=Field isolates showing genus *Staphylococcus* and *mec*A gene-specific bands; L6: Negative control; M: Marker.

Out of 666 samples, a total of 762 Gram-positive bacteria presumptive to be *Staphylococcus* were recovered by the conventional culture method. Duplex PCR detected 728 isolates as *Staphylococcus* spp. and the majority were detected from milk samples (n=451) followed by nasal (n=111), and extramammary sites (n=75) (Table-2). Duplex PCR identified 66 *Staphylococcus* isolates harboring *mec*A gene (9%). The majority of the *mec*A positive isolates were recovered from milk (n=48) followed by animal handlers hand swabs (n=8) (Figure-1). The *mec*A gene was not detected in any of the environmental specimens. All the *Staphylococcus* isolates were negative for *mec*C gene.

**Table-2:** Details of samples collected from various sources.

Source	Number of samples	Number of *Staphylococcus*	Number of *mec*A positive *Staphylococcus*
Milk	371	451	48 (11%)
Nasal	109	111	2 (2%)
Extramammary site	90	75	5 (7%)
Wound	30	28	3 (11%)
Animal handlers hand swab	32	34	8 (24%)
Environmental swabs	34	29	0
Total	666	728	66 (9%)

### Species-specific identification of *Staphylococcus* by multiplex PCR and partial 16S rRNA gene sequencing

**Figure-2:**
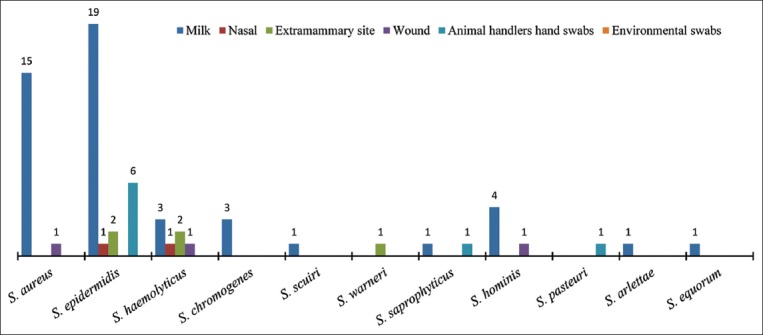
Species diversity of methicillin-resistant *Staphylococcus* isolates recovered from various sample sources.

The 66 *mec*A positive isolates were distributed into five different species by multiplex PCR, of which *S. epidermidis* (n=28) was the most predominant species subsequently proceeded by *S. aureus* (n=16), *S. haemolyticus* (n=7), *S. chromogenes* (n=3), and *S. sciuri* (n=1). The remaining 11 *mec*A positive isolates were unidentified by multiplex PCR (Figure-2). Partial 16S rRNA gene sequence analysis identified 11 unidentified *mec*A positive isolates, namely, five as *S. hominis*, two as *S. saprophyticus*, and one each as *S. warneri*, *S. pasteuri*, *S. arlettae*, and *S. equorum* (Figure-2).

### Molecular characterization by *SCCmec* typing and MLST

SCC*mec* typing of 66 *mec*A positive *Staphylococcus* spp. identified 31 isolates as Type V, whereas the remaining 35 *mec*A positive isolates were non-typeable. The distribution of SCC*mec* types among various species of *Staphylococcus* is detailed in Table-3.

**Table-3:** Distribution of SCCmec types among MRSA and MRCoNS.

Staphylococcus spp.	Source	Type V	Non-typeable
*S. epidermidis*	Milk	8	11
	Nasal	0	1
	Extramammary site	2	0
	Animal handlers hand swab	3	3
*S. aureus*	Milk	12	3
	Wound	1	0
*S. chromogenes*	Milk	2	1
*S. sciuri*	Milk	0	1
*S. haemolyticus*	Milk	0	4
	Nasal	1	0
	Extramammary site	2	0
*S. saprophyticus*	Milk	0	1
	Animal handlers hand swab	0	1
*S. warneri*	Extramammary site	0	1
*S. hominis*	Milk	0	4
	Wound	0	1
*S. pasteuri*	Animal handlers hand swab	0	1
*S. equorum*	Milk	0	1
*S. arlettae*	Milk	0	1
Total (66)		31 (47%)	35 (53%)

*S. epidermidis=Staphylococcus epidermidis, S. aureus=Staphylococcus aureus, S. chromogenes=Staphylococcus chromogenes, S. sciuri=Staphylococcus sciuri, S. haemolyticus=Staphylococcus haemolyticus, S. saprophyticus=Staphylococcus saprophyticus, S. warneri=Staphylococcus warneri, S. hominis=Staphylococcus hominis, S. pasteuri=Staphylococcus pasteuri, S. equorum=Staphylococcus equorum, S. arlettae=Staphylococcus arlettae,* MRSA=Methicillin-resistant Staphylococcus aureus, MRCoNS=Methicillin-resistant coagulase-negative staphylococci

MLST analysis revealed ST 1687 (50%, 8/16) as the predominant ST. Further, six MRSA isolates were found to be novel STs, namely, ST 5217 (n=2), and one each as ST 5216, ST 5218, ST 5219, and ST 5220. Among MRSE, the most predominant ST was found to be ST 457 (14.3%, 4/28) followed by ST 575 (7.14%, 2/28). The other STs identified were ST 439, ST 110, ST 21, ST 226, ST 210, ST 114, ST 130, and ST 57. In addition, 14 MRSE isolates were distributed among nine novel STs, namely, ST 849 (n=3), ST 855 (n=3), ST 854 (n=2), and one each as ST 850, ST 851, ST 852, ST 853, ST 856, and ST 857 (Table-4).

**Table-4:** MLST analysis of methicillin-resistant *S. aureus* and methicillin-resistant *S. epidermidis.*

MRSA/MRSE strains with ST	Source	Number of isolates
MRSA-ST 5216 (novel ST)	Cattle milk	1
MRSA-ST 5217 (novel ST)	Cattle milk	2
MRSA-ST 5218 (novel ST)	Cattle milk	1
MRSA- ST 5219 (novel ST)	Cattle milk	1
MRSA-ST 5220 (novel ST)	Cattle wound	1
MRSA-ST 1687	Cattle milk	8
MRSA –ST 3881	Cattle milk	1
MRSA-ST 2668	Cattle milk	1
MRSE-ST 849 (novel ST)	Cattle milk	3
MRSE-ST 850 (novel ST)	Animal handler’s hand swab	1
MRSE- ST 851 (novel ST)	Animal handler’s hand swab	1
MRSE-ST 852 (novel ST)	Animal handler’s hand swab	1
MRSE-ST 853 (novel ST)	Animal handler’s hand swab	1
MRSE-ST 854 (novel ST)	Cattle milk	1
	Cattle extramammary site	1
MRSE-ST 855 (novel ST)	Cattle milk	3
MRSE-ST 856 (novel ST)	Cattle milk	1
MRSE-ST 857 (novel ST)	Cattle milk	1
MRSE-ST 439	Cattle milk	1
MRSE-ST 110	Cattle milk	1
MRSE-ST 21	Animal handler’s hand swab	1
MRSE-ST 226	Cattle milk	1
MRSE-ST 457	Cattle milk	3
	Cattle extramammary site	1
MRSE-ST 210	Cattle milk	1
MRSE-ST 114	Animal handler’s hand swab	1
MRSE-ST 130	Cattle nasal	1
MRSE-ST 575	Cattle milk	2
MRSE-ST 57 Total	Cattle milk	1 44

MRSA=Methicillin-resistant *Staphylococcus aureus*, MRCoNS=Methicillin-resistant coagulase-negative staphylococci, MLST=Multilocus sequence typing, *S. epidermidis=Staphylococcus epidermidis*, *S. aureus=Staphylococcus aureus*, MRSE-ST=Methicillin-resistant *Staphylococcus epidermidis*-sequence types

## Discussion

Methicillin-resistant *Staphylococcus* is an important pathogen which presently is receiving significant attention in the public and animal health sector. Recognition and discrimination of MRSA and MRCoNS is pre-requisite for implementing appropriate antimicrobial therapy and thereby controlling the advancement of the disease. The current study communicates on the occurrence and characteristics of MRSA and MRCoNS in cattle, cattle handlers, and their environment.

In the present study, the overall detection of methicillin-resistant staphylococci was found to be 9%. The *mec*A gene was observed in 16 isolates (2%) of *S. aureus* strains and 50 isolates of CoNS (7%). The rate of *mec*A positive isolates identified in the present study was low when compared to the other Indian studies, wherein methicillin resistance is recorded between 18 and 35% [26]. One of the reasons for the high rate of methicillin resistance in these studies could be the predominant inclusion of clinical samples. The incidence of MRSA and MRCoNS varied in various countries. In Turkey, Denmark, and China, the frequency of *mec*A positive *Staphylococcus* in humans was found to be 44%, 51%, and 60%, respectively [27-29]. The recovery of *mec*A gene (9%) among our study isolates should be considered a probable risk for public health, as these pathogens may gain access to the food chain. For this reason, it is mandatory to monitor the health status of animals and humans and expand to the hygienic environment. The presence of *mec*A in CoNS is obvious from the study, indicating the crucial role of CoNS in dissemination of methicillin resistance in the environment.

Recently, it has been reported that animals are often colonized with methicillin-resistant staphylococci; particularly, livestock has been a cause of worry, as it has exposed an expanded pool of methicillin resistance [30]. In the present study, 12% (n=8/66) and 88% (n=58/66) of *mec*A positive isolates were obtained from animal handlers and cattle population, correspondingly. Owing to close association of the animals with ecological microbiome and resistome, animal origin staphylococcal strains may serve as a dissemination source of resistance determinants. It has been reported that methicillin resistance can be transmitted to humans either by direct contact with animals, environmental contamination or by handling of products from infected animals [31]. Moreover, companion animals (cats and dogs) are also assumed to acquire resistance from humans [32]. Thus, animals and humans are frequently colonized and both can act as reservoirs of methicillin resistance. Studies have shown that the spread of animal origin MRSA to veterinary personnel is more usual for large animal handlers than small animal handlers [33]. European institutions have played an important role in laying down and passing laws specific to the veterinary sanitary and food safety area for the eradication of certain infectious diseases of livestock [34-37]. The judicial enforcement of these laws will help to prevent the spread of bacterial population from animals to animal handlers or vice versa.

Precise estimations of the effect, sources, transmission dynamics, and control strategies for MRSA/MRCoNS necessitate the exact identification of species. The 66 *mec*A positive staphylococcal isolates identified in the present study were distributed into 11 different species with MRSA representing 24% of the total isolates. These findings are in concordance with the Prasanth *et al*. study [38]; wherein, the authors recorded 29% of the strains from bovine origin as MRSA. Methicillin resistance on an average is high in Indian dairy sectors probably due to lurking of methicillin-resistant genes in the dairy environment [38]. Independent studies from Saudi Arabia [39] and Iran [40] reported 56-57% of MRSA from farm animals and human clinical cases. The authors suggested that the higher prevalence of MRSA in these studies may be due to the inappropriate use of methicillin, which aggravates the dissemination potential between humans and animals as well as to the community. Antoci *et al*. [41] from Southeastern Sicily reported 36% of humans, 61% of cattle, and 44% of milk samples positive for MRSA. In the current study, among MRCoNS, *S*. *epidermidis* (42%), and *S. haemolyticus* (11%) were the most predominant species identified. Huber *et al*. [42] reported 48% of samples from livestock and chicken carcasses positive for MRCoNS with *S. sciuri* (63%) and *S. fleurettii* (17%) representing the prime species. Sawanth *et al*. [43] and Jaglic *et al*. [44] found 30% and 50% of *S. epidermidis* strains harboring the *mec*A gene, respectively, from bovine milk samples. The high prevalence of CoNS may be attributed to the wide distribution of the organism inside the mammary gland and in the teats of the udder. In view of animal contact persons exclusively, we observed *S. epidermidis* (75%, n=6/8) as the most common species. A study from Mexico extended to human sector, identified *S. epidermidis* and *S. haemolyticus* as the most prevalent species among MRCoNS [45]. Similarly, results were obtained from human patients in Algeria, Mali, Moldova, and Cambodia in which *S. epidermidis* and *S. haemolyticus* comprised 98% of the MRCoNS [46]. Cattle and cattle handlers may share *S. epidermidis* strains, implying that bovine MRSE might be a zoonotic pathogen. It is hard to decipher the direction of interspecies transmission; however, it is suggested that *S. epidermidis* will probably spread from humans to dairy cows than the other way around [47].

SCC*mec* elements are genomic islands incorporated into the specific region of the *Staphylococcus* chromosome. In the present study, SCC*mec* type was assigned to 47% (n=31) of *mec*A positive isolates. SCC*mec* Type V was identified among 81% of MRSA, whereas among MRCoNS, 26% of *S. epidermidis*, 6% of *S. haemolyticus*, and 4% of *S. chromogenes* belonged to Type V. According to Ruppe *et al*. [46], SCC*mec* Type IV predominates in *S. epidermidis*, while Type V predominates in *S. haemolyticus* and Type III in *S. aureu*s and variety of CoNS. Our observations are in align with the Fessler *et al*. study [48]; wherein, all the methicillin-resistant *S. haemolyticus* of bovine mastitis origin was identified with SCC*mec* element Type V. Detection of SCC *mec* Type V (which is considered as a characteristic feature of CA-MRSA) among MRSA and MRCoNS belonging to the same farm supports the hypothesis for the possible transfer of SCC*mec* between CoNS and *S. aureus*. The increasing incidence of SCC*mec* type in certain CoNS species should be analyzed with caution. Studies have demonstrated that other than the prevailing SCC*mec* type, various other types may appear in members of the CoNS species. We observed 35 *mec*A positive isolates non-typeable for SCC*mec* element. The possible explanation could be either *ccr* genes might be anonymous types or may have undergone certain mutations in the primer-target sites or presumably lost [49]. SCC*mec* typing in MRCoNS is challenging due to its current identification of co-existed SCC*mec* and the existence of non-typeable components [7]. In spite of the fact that it may be hard to detect all SCC*mec* types carried by *Staphylococcus* spp, interpreting as many SCC*mec* types as possible will further help for epidemiological studies and in outlining the sources of MRSA/MRCoNS strains.

Among MRSA isolates, we identified ST 1687 as the most common ST which was detected earlier by Mistry *et al*. [50] as the novel ST type among milk samples obtained from mastitis affected cows of Telangana and Tamil Nadu regions from India. ST 2668 detected in our study among MRSA isolates were also previously reported in 3.5% of MSSA clones among environmental samples of Chinese Metro systems [51]. The study demonstrated that environmental surfaces may be a hazardous reservoir for transmission of methicillin-resistant *Staphylococci* to passengers. Cross transmissions of MRSA/MRCoNS isolates from various sources, including hospitals, communities, and livestock, are also possible. Thus, more stringent infection control and surveillance measures are needed. Moreover, we identified ST 457 as the most prevalent ST among MRSE. Previously, Armand-Lefevre *et al*. [52] reported ST 457 as the novel ST among pig farmers. The occurrence of the same STs among pig farmers and cattle probably suggests the host jump/adaptation and clonal evolution of the strains which further creates great havoc. The significance of this study is the detection of new STs, which will permit further analyses to keep pace with new evolutionary trends. Moreover, a high proportion of new STs among human population who were in close association with cattle may be explained by transmission of resistant determinants between humans and animals and vice versa. Further, whole-genome sequencing of these isolates will mark recognition of genetically closely related isolates within the STs, thereby tracing out the potential sources and identifying outbreaks.

## Conclusion

The study highlights a high prevalence of methicillin resistance in the dairy environment with *S. epidermidis* as the most leading species. Prevalence of methicillin resistance among animal handlers was found to be 24% which was very high as compared to 8.7% in cattle. Thus, individuals with persistent animal contact should be educated on the risk of probable transmission of resistance from animals to humans and vice versa. As the transmission of resistant genes is dynamic and involves animals, humans, and their environment, it necessitates periodic surveillance of the resistance status of *S. aureus* and CoNS to control the spread of resistance and reduce disease burden associated with these resistant pathogens. Further, genomic characterization to find resistance level variations is essential to interpret human and animal transmission dynamics.

## Authors’ Contributions

BRS supervised the group and executed the project, and contributed to the drafting and revision of the manuscript. RS and HR helped in the design of sample collection strategies and critical revision of the manuscript. NV collected samples, designed, and performed experiments, data analysis and contributed to the drafting and revision of the manuscript. SM and RT collected data, performed experiments, and critical revision of the manuscript. FG carried out data analysis and interpretation and contribution to drafting and critical revision of the manuscript. All authors read and approved the final manuscript.
